# FGFR3 mRNA overexpression defines a subset of oligometastatic colorectal cancers with worse prognosis

**DOI:** 10.18632/oncotarget.25941

**Published:** 2018-08-14

**Authors:** Julia Elisabeth Fromme, Katja Schmitz, Astrid Wachter, Marius Grzelinski, Dirk Zielinski, Christina Koppel, Lena-Christin Conradi, Kia Homayounfar, Tabea Hugo, Sara Hugo, Laura Lukat, Josef Rüschoff, Philipp Ströbel, Michael Ghadimi, Tim Beißbarth, Kirsten Reuter-Jessen, Annalen Bleckmann, Hans-Ulrich Schildhaus

**Affiliations:** ^1^ Institute of Pathology, University Hospital Göttingen, Göttingen, Germany; ^2^ Department of Medical Statistics, University Medical Center Göttingen, Göttingen, Germany; ^3^ Targos Molecular Pathology Inc., Kassel, Germany; ^4^ Department of General, Visceral and Pediatric Surgery, University Medical Center Göttingen, Georg-August-University, Goettingen, Germany; ^5^ Department for Hematology and Medical Oncology, University Hospital Göttingen, Göttingen, Germany

**Keywords:** colorectal cancer, metastases, FGFR3, fibroblast growth factor receptors, RNA in situ hybridization

## Abstract

**Objectives:**

Metastatic colorectal cancer (CRC) remains a leading cause of cancer related deaths. Patients with oligometastatic liver disease represent a clinical subgroup with heterogeneous course. Until now, biomarkers to characterize outcome and therapeutic options have not been fully established.

**Methods:**

We investigated the prevalence of FGFR alterations in a total of 140 primary colorectal tumors and 63 liver metastases of 55 oligometastatic CRC patients. FGF receptors (*FGFR1-4*) and their ligands (*FGF3, 4* and *19*) were analyzed for gene amplifications and rearrangements as well as for RNA overexpression *in situ*. Results were correlated with clinico-pathologic data and molecular subtypes.

**Results:**

Primary tumors showed *FGFR1* (6.3%) and *FGF3,4,19* (2.2%) amplifications as well as FGFR1 (10.1%), FGFR2 (5.5%) and FGFR3 (16.2%) overexpression. In metastases, we observed *FGFR1* amplifications (4.8%) as well as FGFR1 (8.5%) and FGFR3 (14.9%) overexpression. Neither *FGFR2-4* amplifications nor gene rearrangements were observed. FGFR3 overexpression was significantly associated with shorter overall survival in metastases (mOS 19.9 vs. 47.4 months, HR=3.14, p=0.0152), but not in primary CRC (HR=1.01, p=0.985). Although rare, also *FGFR1* amplification was indicative of worse outcome (mOS 12.6 vs. 47.4 months, HR=8.83, p=0.00111).

**Conclusions:**

We provide the so far most comprehensive analysis of FGFR alterations in primary and metastatic CRC. We describe FGFR3 overexpression in 15% of CRC patients with oligometastatic liver disease as a prognosticator for poor outcome. Recently FGFR3 overexpression has been shown to be a potential therapeutic target. Therefore, we suggest focusing on this subgroup in upcoming clinical trials with FGFR-targeted therapies.

## INTRODUCTION

The fibroblast growth factor receptor (FGFR) family comprises four tyrosine kinase receptors (FGFR1-4). The FGFR pathway mediates basic processes in embryogenesis and plays an important role for cellular proliferation, differentiation and angiogenesis in adult tissue [1, 2]. The receptors are activated by 18 FGF ligands acting in a paracrine or endocrine way [1–4]. The ligands bind to heparan sulfate proteoglycans (HPSGs) on the cell surface consolidating the ligand-receptor bond [5]. Specific binding is ensured by means of alternative splicing of the FGFRs, ligand-receptor specificity [6, 7] or by Klotho proteins as co-factors which facilitate the ligand-receptor interaction in the context of endocrine stimulation [8]. After receptor dimerization and autophosphorylation of the cytoplasmic domain the tyrosine kinase interacts with adapter proteins which activate downstream signaling pathways as RAS-RAF-MAPK, STAT and PI3K-AKT [1, 2]. Recently, different types of FGFR alterations have been found in various cancer entities comprising gene amplifications, translocations, gain of function mutations, ligand-dependent activation or overexpression of FGFRs and FGFs [1, 9, 10]. *FGFR1* amplification is present in 20% of squamous cell lung cancer,[11, 12] 5% of small cell lung cancer [13] and in 10% of breast cancers,[14, 15] *FGFR2* amplifications or mutations in about 5% of gastric cancer,[16] *FGFR3* mutations in 10-15% of muscle invasive bladder cancer [17, 18]. *FGFR3* translocations occur in 3-7% of glioblastoma [19, 20] and in 15-20% of myelomas [21, 22]. In these entities, FGFR signaling pathway alterations can drive oncogenesis by excessive cell proliferation, migration, neovascularization and thus have a negative prognostic impact [2].

Beside aberrations in the FGFRs, also FGF ligand alterations play a decisive role. Especially the FGF ligands 3, 4 and 19 have been suggested to be of particular importance. Parish et al. reported on a co-amplification of *FGF3,4* and *19* in 5.6% of cancers [23]. Amplification of *FGF3* has been described in 15-20% of breast cancers and is associated with a more rapid tumor progression [24]. In most cases, *FGF3,4* and *19* are co-amplified since they are localized next to each other in one cluster on the long arm of chromosome 11 (11q13). FGF19 is essential for FGFR1 (and FGFR4) signaling [25].

Given the high prevalence of FGF/FGFR aberrations in advanced cancers and in view of its prognostic impact, numerous selective and unselective FGFR inhibitors are currently being tested in phase I and II clinical trials. The multikinase inhibitor nintedanib, an anti-angiogenic inhibitor of VEGFR and PDGFR with anti-FGFR activity, in combination with chemotherapy has shown to be non inferior to bevacizumab-based regimen in metastatic colorectal cancer in a phase II clinical trial. Furthermore, even mCRC patients who were extensively pretreated benefited from nintedanib in terms of longer progression free survival and better quality of life [26]. Several additional multikinase inhibitors such as lucitanib (active against VEGFR1-3, PDGFRα/β and FGFR1-3), lenvatinib (E7080, Eisai; an inhibitor of FGFR, VEGFR, PDGFR, RET and KIT) or dovitinib (FGFR, VEGFR, PDGFR, CSF-1 and c-kit inhibitor) showed promising response rates in several phase I/II trials in breast cancer, solid tumors and renal carcinomas, respectively [27–29].

Selective FGFR inhibitors, however, are thought to target FGFRs even more specifically while causing less side-effects. In *FGFR3* mutant bladder/urothelial cancer and *FGFR1* amplified NSCLC the selective FGFR1-3 tyrosine kinase inhibitor BGJ398 has shown partial responses and was well tolerated [30]. Anti-tumor effects were also recognized in cholangiocarcinoma harboring a *FGFR2* fusion and in *FGFR1* amplified breast cancer [31]. Another potent selective FGFR inhibitor is AZD4547, which has been tested in a phase II clinical trial for patients with advanced breast, lung and gastric cancer harboring *FGFR1* or *FGFR2* amplifications. Published response rates were 33% in *FGFR2* amplified gastroesophageal cancer and 12.5% in *FGFR1* amplified breast cancer [32]. The pan-FGFR inhibitor JNJ-42756493 showed partial responses in tumors which harbored a translocation of *FGFR2* or *FGFR3* [33]. In the phase I study with the pan-FGFR inhibitor BAY1163877 seven patients (87.5%) with urothelial bladder cancer experienced a tumor shrinkage [34].

*FGFR* or *FGF 3,4,19* (11q) genomic amplification, gene fusions or mutations have been used as biomarkers in most clinical trials so far [30, 35, 36]. Some of these trials could demonstrate a correlation between therapeutic response and genomic changes such as amplification measured by fluorescence *in situ* hybridization (FISH). In contrast, in the phase I study of the pan-FGFR inhibitor BAY1163877 elevated mRNA expression levels of FGFR1-3 analyzed by RNA *in situ* hybridization were proposed for selection of patients [34]. The majority of tumors which responded showed elevated FGFR mRNA levels but no genomic FGFR alteration [34]. Also in lung cancer and head and neck cancer (treated with BGJ 398) mRNA expression level has been suggested as a potential predictor for anti-FGFR effects [37, 38].

Up to now published data on FGFR alterations in colorectal cancer is very sparse. However, there is a clinical need for targeted treatments. When colorectal cancer is diagnosed in a metastatic state, almost 90% of the patients die within five years after diagnosis (based on SEER DataBase). The introduction of anti-EGFR therapy for *RAS* wildtype CRC, the anti-angiogenic therapy with VEGF antibodies and immune checkpoint inhibitors for microsatellite instable cancers have improved systemic CRC therapy in recent years. However, there is still a significant percentage of metastatic patients who are not eligible for these treatments or do not respond. Thus, the aim of our study was i) to comprehensively analyze FGFR alterations in metastatic and primary CRC, ii) to investigate the prevalence of the potential predictive biomarkers and iii) to analyze their prognostic relevance.

In this study, we focused on oligometastatic CRC, i.e. on patients with a limited number of resectable liver metastases. These patients form a clinically meaningful but heterogeneous group in terms of prognosis. Many of them can be cured by surgery alone but others progress rapidly and need a continuation of systemic treatment. There is a specific clinical need for prognostic markers in these patients. Moreover, personalized treatment options may help to improve therapy for those patients who are at high risk.

We comprehensively investigated alterations at the genomic and expression levels of FGF/FGFR and compared these findings with data from primary tumors. For the first time, we systematically report on prevalence data of predictive biomarkers which may provide the basis for upcoming clinical trials with anti-FGFR drugs in patients with metastatic colorectal cancer. Moreover, we demonstrate that FGFR3 overexpression defines a clinically highly significant subgroup with worse prognosis. We suggest including particularly these patients in ongoing and upcoming clinical trials with anti-FGFR compounds.

## RESULTS

### RNA overexpression of FGFR1-3

**Figure 1:**
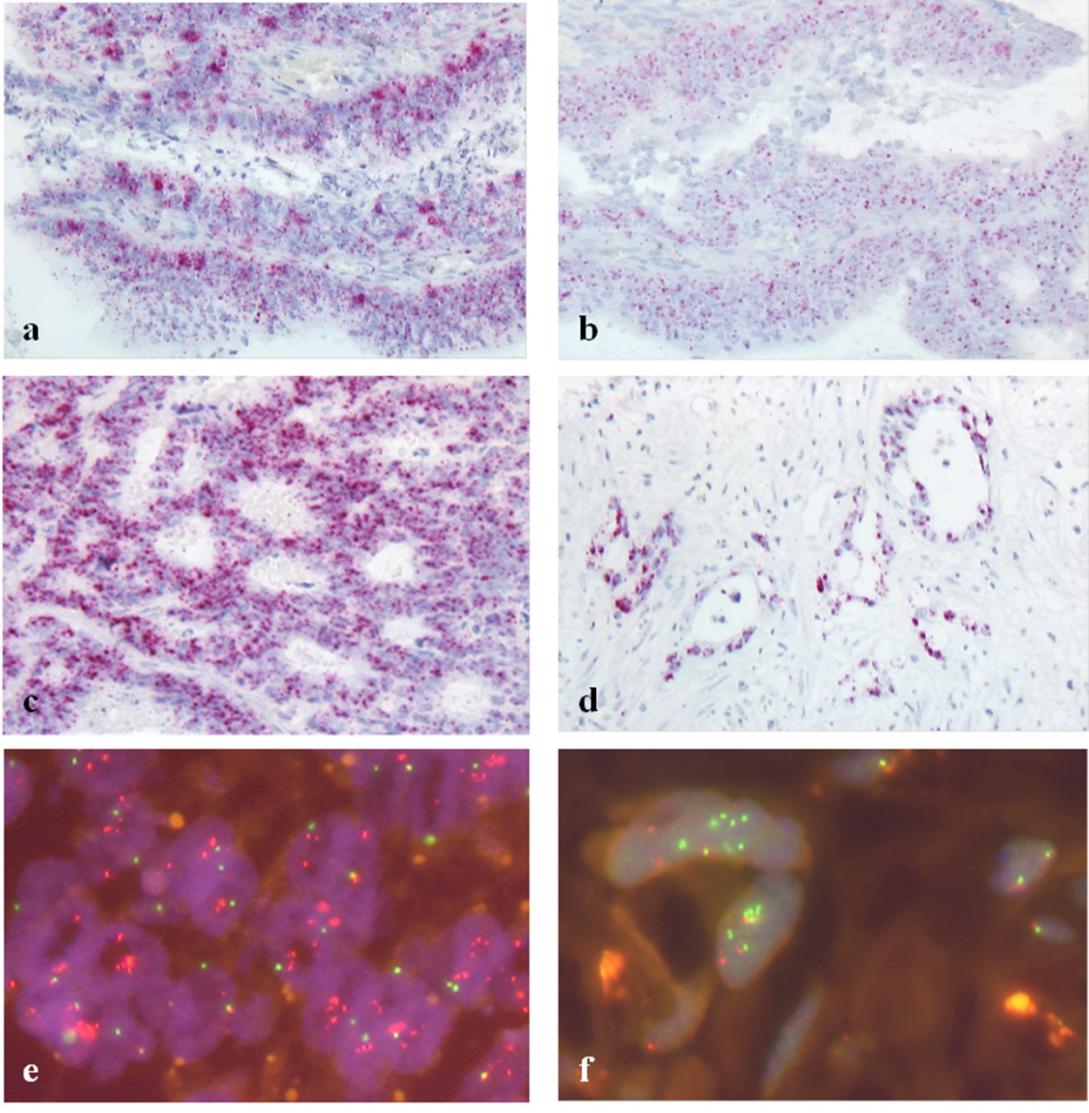
Fibroblast growth factor receptor (FGFR) overexpression (RNA ISH, a-d) and *FGFR* gene amplification (FISH, e-f) (a+b) FGFR1 **(a)** and FGFR2 **(b)** overexpressing colon cancer; H-score 250 and 220, respectively; predominant score 3 for both receptors; **(c)** FGFR3 overexpressing liver metastasis; H-score 350, predominant score 4; **(d)** FGFR3 overexpressing liver metastasis; H-score 360, predominant score 4; **(e)** *FGF3,4,19* high-level amplified rectal cancer, **(f)** *FGFR1* high-level amplification in a liver metastasis.

FGFR1 overexpression was found in 8.5% (four out of 47 evaluable cases) of the liver metastases (Table 1) and in 10.1% (10/99) of the evaluable primary tumors (colon n=7, rectum n=3). FGFR2 overexpression occurred in none of the metastases of our cohort, but in 5.5% (5/91) of the primary tumors (colon n=4, rectum n=1). FGFR3 mRNA overexpression was the most frequent finding across all tissues. 14.9% of the liver metastases showed FGFR3 overexpression (7/47, H-score 200-360, Figure 1). Among the primary tumors FGFR3 mRNA overexpression occurred in 16.2% of cases (18/111; colon n=11, rectum n=7).

**Table 1:** FGFR mRNA overexpressing cases, molecular subtypes, gene amplification and clinical data

No.						RNA ISH		FISH			
	Receptor	Tumor type	*RAS*	*PIK3CA*	*BRAF*	H-score	Pred. score	Ratio	Average *FGF(R)* signals (%)	Cells with ≥5 *FGF(R)* signals (%)	Amplification level
1	FGFR1	C	Mt	--	--	250	2	0.6	1.3	1.7	neg (*FGFR1*)
2	FGFR1	C	--	--	--	310	3	1.0	2.2	1.7	neg (*FGFR1*)
3	FGFR1	R	WT	--	--	230	2	--	--	--	--
4	FGFR1	LM/C	Mt	WT	WT	200	2	1.0	2.1	0	neg (*FGFR1*)
5	FGFR1	LM/C	Mt	WT	WT	210	2	1.0	1.9	0	neg (*FGFR1*)
6	FGFR1	LM/C	Mt	WT	WT	210	2	1.1	2.2	0	neg (*FGFR1*)
7	FGFR1	R	Mt	--	--	230	2	0.9	1.7	0	neg (*FGFR1*)
8	FGFR1	C	WT	--	--	220	2	--	--	--	--
9	FGFR1	LM/R	Mt	WT	WT	260	3	0.9	1.9	1.7	neg (*FGFR1*)
10	FGFR2	R	WT	--	--	310	4	1.1	2.6	8.3	neg (*FGFR2*)
11	FGFR2	C	WT	--	--	200	2	1.0	1.9	0	neg (*FGFR2*)
12	FGFR3	R	WT	--	--	260	4	1.0	1.7	0	neg (*FGFR3*)
13	FGFR3	LM/R	WT	WT	WT	325	4	1.2	2.1	0	neg (*FGFR3*)
14	FGFR3	R	WT	--	--	260	4	1.2	2.0	0	neg (*FGFR3*)
15	FGFR3	C	WT	--	--	280	3	1.0	1.7	2.0	neg (*FGFR3*)
16	FGFR3	C	WT	--	--	200	2	1.1	1.7	0	neg (*FGFR3*)
17	FGFR3	C	Mt	--	--	260	2	1.1	2.2	0	neg (*FGFR3*)
18	FGFR3	C	--	--	--	240	2	--	--	--	--
19	FGFR3	LM/R	WT	WT	WT	210	2	1.2	2.2	6.7	neg (*FGFR3*)
20	FGFR3	R	WT	--	--	340	4	1.0	1.7	0	neg (*FGFR3*)
21	FGFR3	LM/C	Mt	WT	WT	340	4	1.1	1.8	0	neg (*FGFR3*)
22	FGFR3	LM/R	WT	WT	WT	350	4	1.0	1.7	0	neg (*FGFR3*)
23	FGFR3	LM/R	Mt	WT	WT	310	4	1.2	1.9	0	neg (*FGFR3*)
24	FGFR3	LM/C	Mt	WT	WT	200	2	1.1	2.2	0	neg (*FGFR3*)
25	FGFR3	R	--	--	--	310	4	0.9	1.9	0	neg (*FGFR3*)
26	FGFR3	C	WT	--	--	300	3	0.9	1.7	0	neg (*FGFR3*)
27	FGFR3	C	WT	--	--	220	3	1.0	1.5	0	neg (*FGFR3*)
28	FGFR1	C	WT	--	--	250	3	1.2 12.9	4.1 29.9	45 100	neg (*FGFR1*) HL (*FGF3,4,19*)
29	FGFR1 FGFR3	C	WT	--	--	220 200	2 2	1.2 1.6	2.9 2.8	5 13.3	neg (*FGFR1*) neg (*FGFR3*)
30	FGFR1 FGFR3	R	--	--	--	210 250	2 4	1.0 1.0	2.4 1.6	1.7 0	neg (*FGFR1*) neg (*FGFR3*)
31	FGFR1 FGFR3	C	WT	--	--	340 230	3 2	1.6 1.1	2.0 2.3	0 2	neg (*FGFR1*) neg (*FGFR3*)
32	FGFR1 FGFR2 FGFR3	C	--	--	--	250 220 210	3 3 2	-- 1.0 0.9	-- 2.8 1.7	-- 8.3 0	-- neg (*FGFR2*) neg (*FGFR3*)
33	FGFR2 FGFR3	C	Mt	--	--	220 200	3 2	1.1 1.1	2.0 2.3	0 0	neg (*FGFR2*) neg (*FGFR3*)
34	FGFR2 FGFR3	C	Mt	--	--	200 200	2 2	1.0 1.1	2.1 2.0	1.7 0	neg (*FGFR2*) neg (*FGFR3*)
35	FGFR3	LM/R	WT	WT	WT	360	4	1.2 2.4	2.3 4.9	3.3 48.3	neg (*FGFR3*) HL (*FGFR1*)
36	FGFR3	R	WT	--	--	310	4	1.1 1.2	2.2 5.5	0 66.7	neg (*FGFR3*) LL (*FGFR1*)
37	FGFR3	R	WT	--	--	310	4	0.9 2.9	1.6 5.1	0 45	neg (*FGFR3*) HL (*FGF3,4,19*)

Overview over all cases with any FGFR mRNA overexpression, based on high RNA ISH levels (cut-off: H-score ≥200). Pred. Score, predominant score; M, male; F, female;

Tumor types: C, primary colonic cancer; R, primary rectal cancer; LM/C, liver metastasis with primary tumor in the colon; LM/R, liver metastasis with primary tumor in the rectum; Pred. score, predominant score; Mt, mutation; WT, wildtype; neg, negative; pos, positive; --, data not available HL, high level; LL, low level.

RNA overexpression was measured by *in situ* hybridization and defined by an H-score ≥200. We also evaluated the predominant score as another criterion for mRNA overexpression (positive cases defined based on the cut-off ≥3). For FGFR3, the two scores correlated highly significantly in metastases (p≤0.001). In primary tumors, both scoring approaches correlated highly significantly for FGFR1, FGFR2 and FGFR3 (p≤0.001).

### Analysis of gene amplification of *FGF* receptors and ligands, gene translocations and cases with multiple aberrations

We found a high-level amplification of *FGFR1* in 4.8% (two out of 42 evaluable cases) among liver metastases (Table 2, Figure 1). One of those cases originated from a rectum cancer, the other from a colon carcinoma. The primary tumor as well as a lymph node metastasis of the latter case could also be investigated. Both of these materials did not formally reach the high level of amplification but were quite close to it (*FGFR1*/CEN8 ratio 1.9; see case no. 4.1, Table 3 , for details). This particular patient had received chemotherapy within 6 months before resection of the metastasis.

**Table 2:** FISH positive cases, corresponding molecular subtypes, RNA ISH and clinical data

No.						FISH				RNA ISH	
	Receptor/ligand	Tumor type	*RAS*	*PIK3CA*	*BRAF*	Ratio	Average *FGF(R)* signals (%)	Cells with ≥5 *FGF(R)* signals (%)	Ampli-fication level	H-score	Result
1	*FGFR1*	C	WT	--	--	1.8	5.7	60	Low	100	neg (FGFR1)
2*(36)	*FGFR1*	R	WT	--	--	1.2	5.5	66.7	Low	101 310	neg (FGFR1) pos (FGFR3)
3	*FGFR1*	R	WT	--	--	3.0	4.7	56.7	High	101	neg (FGFR1)
4	*FGFR1*	R	Mt	--	--	4.4	11.4	95	High	180	neg (FGFR1)
5	*FGFR1*	R	WT	--	--	2.1	3.2	33.3	High	101	neg (FGFR1)
6	*FGFR1*	LM/C	WT	WT	WT	2.0	3.6	35.0	High	105	neg (FGFR1)
7*(35)	*FGFR1*	LM/R	WT	WT	WT	2.4	4.9	48.3	High	135 360	neg (FGFR1) pos (FGFR3)
8*(37)	*FGF3,4,19*	R	WT	--	--	2.9	5.1	45.0	High	310	pos (FGFR3)
9*(28)	*FGF3,4,19*	C	WT	--	--	12.9	29.9	100	High	250	pos (FGFR1)

^*^(X), number of the same case in Table 1 ; M, male; F, female; tumor types: C, primary colonic cancer; R, primary rectal cancer; LM/C, liver metastasis with primary tumor in the colon; LM/R, liver metastasis with primary tumor in the rectum; Mt, mutation; WT, wildtype; neg, negative; pos, positive; --, data not available.

**Table 3:** Patients with multiple synchronous or metachronous metastases, cases where multiple tumor manifestations were investigated

No.			RNA overexpression			Gene amplification	Molecular subtyping
	Origin of primary tumor	Time period from first metastasis to subsequent metastases (months)	FGFR1 (result)	FGFR2 (result)	FGFR3 (result)	*FGFR1* Ratio/GCN (amplification level)	*RAS/BRAF/PIK3CA*
**1.1**	R	0	0 (neg)	100 (neg)	160 (neg)	1.2/2.4 (neg)	WT WT WT
**1.2**		8	100 (neg)	45 (neg)	140 (neg)	1.2/2.5 (neg)	WT WT WT
**1.3**		10	120 (neg)	100 (neg)	320 (pos)	1.2/2.5 (neg)	WT WT WT
**1.4**		10	100 (neg)	100 (neg)	260 (pos)	1.1/2.1 (neg)	WT WT WT
1.5*(22)		10	20 (neg)	100 (neg)	350 (pos)	1.3/2.5 (neg)	WT WT WT
**2.1**	R	0	NA	NA	NA	1.1/2.0 (neg)	WT WT WT
**2.2**		0	NA	100 (neg)	140 (neg)	1.0/1.8 (neg)	WT WT WT
**2.3**		0	NA	NA	NA	1.2/2.3 (neg)	WT WT WT
**2.4**		0	NA	100 (neg)	100 (neg)	1.0/2.0 (neg)	WT WT WT
**3.1**	C+R^1^	0	105 (neg)	110 (neg)	130 (neg)	0.5/1.2 (neg)	Mt WT WT
**3.2**		NA	0 (neg)	130 (neg)	140 (neg)	0.5/1.1 (neg)	Mt WT WT
**4.1**^2^**§(6)**	C	0	105 (neg)	100 (neg)	130 (neg)	2.0/3.6 (high)	WT WT WT

^*^(X), number of the same case in Table 1 ; §(X), number of the same case in Table 2 ; C, colon; R, rectum; WT, wildtype; Mt, mutation; GCN, average gene copy number; neg, negative; pos, positive; NA, not available.

^1^This patient had a colon (cecum) and a rectal carcinoma simultaneously, hence it remains unclear, which carcinoma caused the metastases.

^2^The primary tumor as well as a lymph node metastasis did not show *FGFR1* high-level amplification, but low-level amplifications (colon carcinoma: *FGFR1* ratio 1.9, avGCN 5.0, cells with ≥5 *FGFR1* signals: 50%; lymph node metastasis: *FGFR1* ratio 1.9, avGCN 5.7, cells with ≥5 *FGFR1* signals: 65%).

6.3% of evaluable primary tumors (5/80) showed an amplification of *FGFR1* and 2.2% harbored a *FGF3,4,19* amplification (2/89). One FGF3,4,19 amplified colon carcinoma showed an extraordinary high amplification pattern with a *FGF-3,4,19*/CEN11 ratio of 12.9 and an average gene copy number of 29.9. Among metastases no *FGF-3,4,19* amplified cases were seen. Neither amplifications of FGFR2, FGFR3 or FGFR4 nor any gene rearrangements of FGFR1 or FGFR3 were observed.

In summary, twelve metastases and 30 primary tumors harbored any alteration in the FGFR pathway regardless of the type of aberration or the affected receptor. For both cohorts FGFR3 overexpression was the most frequent aberration in this context.

**Figure 2:**
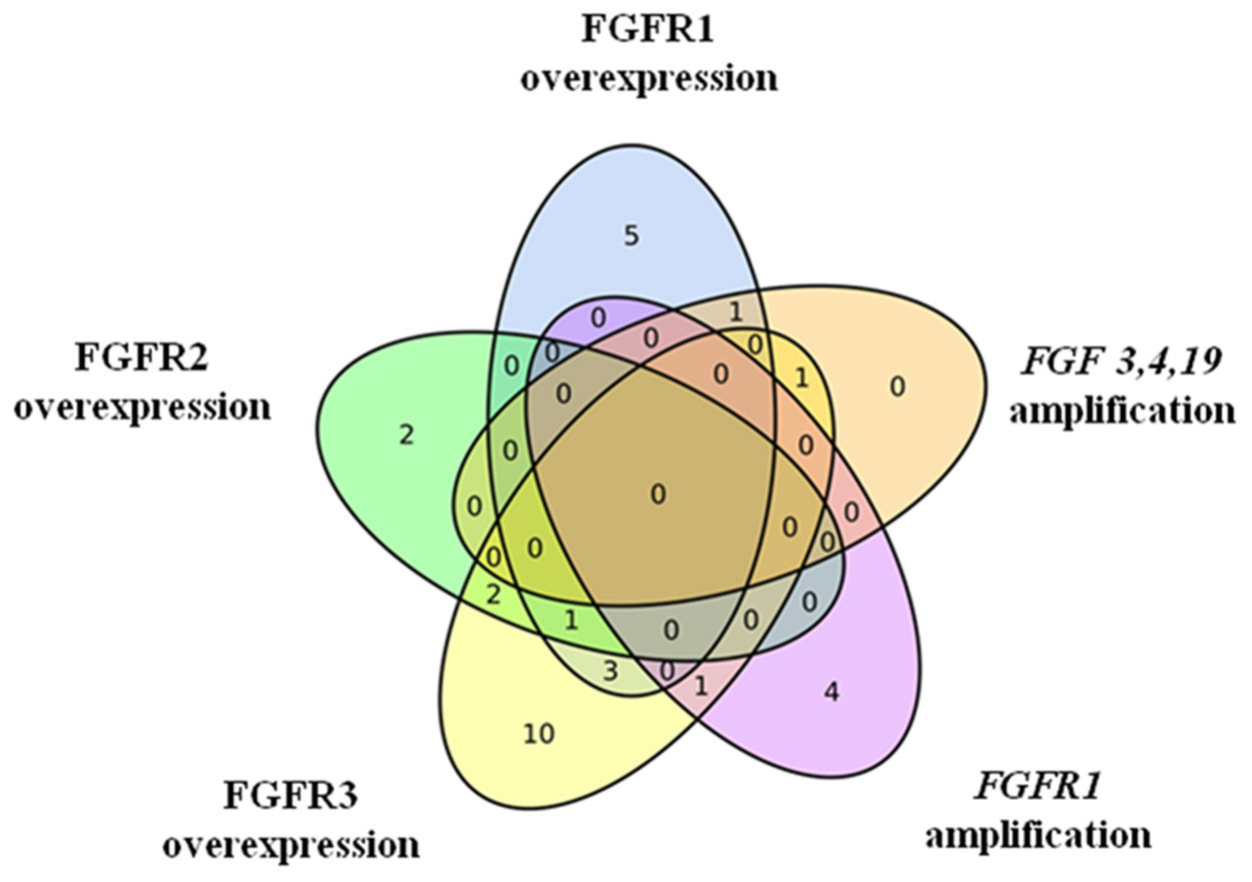
FGFR aberrations in primary tumors Venn diagram shows that many tumors harbor multiple alterations but FGFR3 overexpression is by far the most common event.

Moreover, we also recognized tumors with multiple FGFR aberrations (one of the liver metastases and 9 primary tumors; among them 6 colon carcinomas, mostly right-sided, n=5, 3 rectal carcinomas; Table 1, Figure 2). *FGF3,4,19* amplification was always associated with mRNA overexpression, either of FGFR1 or FGFR3*. FGFR1* amplification appeared either independently or in two cases in association with FGFR3 overexpression. Overexpression of more than one type of FGFR mRNA occurred in various cases.

We could investigate more than one metastasis of three patients. One of these patients (patient 1 in Table 3 ) underwent subsequent resection of five metastases. Interestingly, only the three metastases that occurred the latest showed an overexpression of FGFR3 whereas the two earlier lesions harbored no aberration in the FGFRs. This might indicate that FGFR alterations might be a late event in tumor progression. The patient had received chemotherapy in combination with EGFR antibodies prior to occurrence of the FGFR3 positive metastases. We did not find significant differences in two more patients with multiple synchronous or metachronous metastatic lesions (Table 3).

### Correlation of amplification and mRNA overexpression with clinico-pathologic data and molecular subtypes

**Figure 3:**
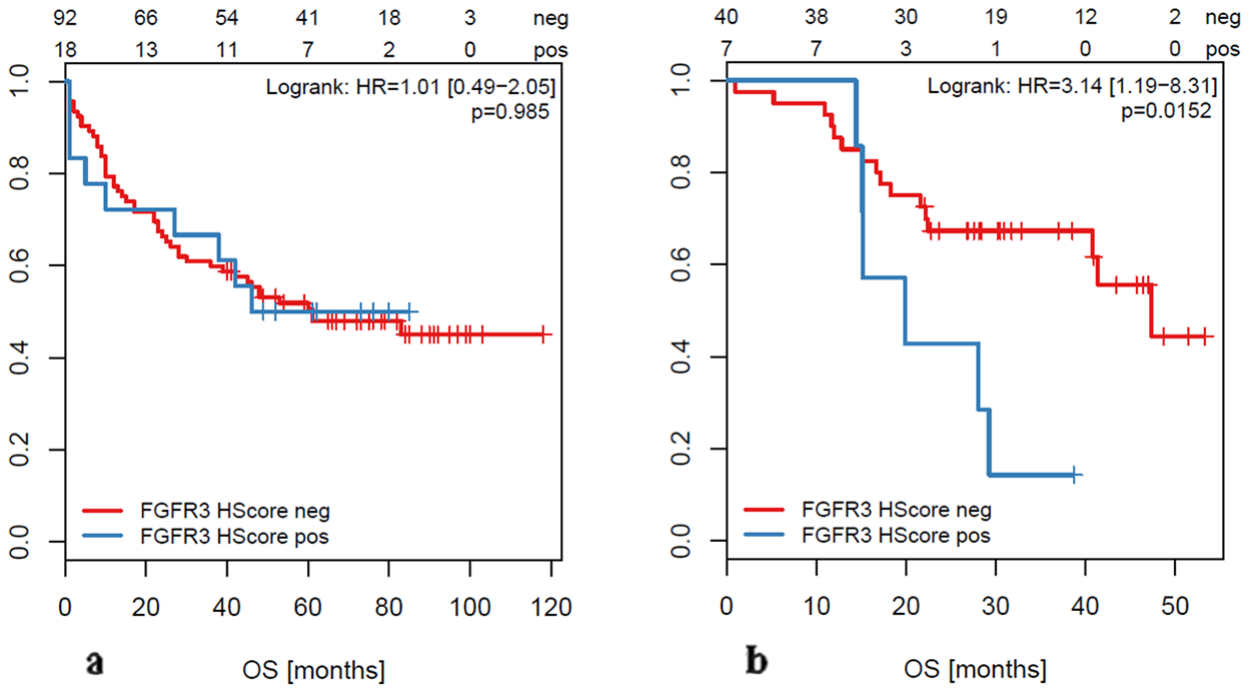
Overall Survival (OS) of patients with FGFR3 overexpression (based on H-score ≥200) measured in primary tumors (a) and in metastases (b)

In metastatic lesions, we found a significant correlation between overall and cancer specific survival and FGFR alterations. FGFR3 mRNA overexpression was significantly associated with reduced overall survival (p=0.0152, HR=3.14 [1.19-8.31]; Figure 3, Table 4 ) and worse cancer specific survival (p=0.00497, HR=3.8 [1.4-10.35]; not shown) in metastasized CRC. In view of FGFR3 as a potential therapeutic option, it is noteworthy that less than 50% of the liver metastases with FGFR3 overexpression were associated with *RAS* mutations (see below). *BRAF* and *PIK3CA* were wildtype in all FGFR3 overexpressing cases.

**Table 4:** Prognostic significance of *FGF/FGFR* gene amplification and FGFR overexpression in primary and oligometastatic CRC

Lesion	Receptor/ ligand	Status	Events/total	Median overall survival in months (95%CI)	Log rank test
Primary tumors					
	***FGFR1***	Amplified	3/5	38 (3-NA)	
		non-amplified	33/73	NA (42-NA)	P= 0.404
	***FGF 3,4,19***	amplified	2/2	11.5 (10-NA)	
		non-amplified	41/85	NA (38-NA)	P= 0.059
	**FGFR1**	Overexpression	7/10	28 (7-NA)	
		no overexpression	47/88	53 (38-NA)	P= 0.228
	**FGFR2**	Overexpression	3/5	46 (42-NA)	
		no overexpression	47/85	53 (28-NA)	P= 0.99
	**FGFR3**	Overexpression	9/18	46 (27-NA)	
		no overexpression	48/92	61 (39-NA)	P= 0.985
Metastases	***FGFR1***	Amplified	2/2	12.6 (5.24-NA)	
		non-amplified	15/40	47.4 (40.79-NA)	**P= 0.00111**
	******FGF 3,4,19******	Amplified	0	NA	
		non-amplified	24/51	41.4 (28.1-NA)	NA
	**FGFR1**	Overexpression	1/4	NA (22.2-NA)	
		no overexpression	21/43	40.8 (28.1-NA)	P= 0.428
	**FGFR2**	Overexpression	0	NA	
		no overexpression	21/45	41.4 (28.1-NA)	NA
	**FGFR3**	Overexpression	6/7	19.9 (15.1-NA)	
		no overexpression	16/40	47.4 (40.8-NA)	**P= 0.0152**

Gene amplification was measured by FISH, overexpression by RNA ISH, based on H-score; CI, confidence interval; NA, not available.

Furthermore, we also recognized an association between *FGFR1* amplification and overall survival (p=0.00111, HR=8.83 [1.82−42.95]) in metastatic patients (Table 4). However, since there were only two *FGFR1* amplified metastases in our cohort, the number of positive cases is too small and larger case numbers would be required to confirm this finding.

For metastases, there was no association between *FGFR* overexpression or amplification and age of the patient, gender, localization of the primary tumor (colon vs. rectum, right-sided vs. left-sided colon), microsatellite instability as well as molecular subtypes.

We did not find any significant association between FGFR alterations and clinico-pathologic data such as age, gender, size or location of the tumor (right-sided vs. left-sided), lymph node or distant metastases, microsatellite instability, *KRAS* mutation and overall survival in primary tumors. In sharp contrast to the cohort of oligo-metastatic CRC, there was no positive correlation between FGFR3 overexpression and survival in primary CRC (p=0.985, HR=1.01 [0.49-2.05]; Figure 3). Among our cohort of primary tumors, there were 33 (23.6%) metastatic cases. Also in this subgroup, there was no correlation between FGFR3 overexpression (measured in the primary tumor) and overall survival (log rank test; p=0.992). Comparing primary tumors with and without metastases in terms of *FGFR* amplification or mRNA overexpression we did not see any significant difference between the two groups.

FGFR3 overexpression occurred against a background of *RAS* wildtype in 57.1% of metastases (Table 1). All of them were wildtype for *BRAF* and *PIK3CA*. The metastases showing FGFR1 overexpression (n=4) were all *RAS* mutated, whereas *BRAF* and *PIK3CA* were wildtype. *FGFR1* amplified liver metastases (n=2) were both *RAS*, *BRAF* and *PIK3CA* wildtype (Table 2). Statistically, we did not find a significant correlation between *FGFR* amplification or overexpression and the mutational status of *RAS*, *BRAF* and *PIK3CA* in the cohort of metastases. Only three primary tumors with FGFR3 overexpression were *RAS* mutated. The percentages of *RAS* mutated cases were 50% and 29% among primary cancer with FGFR2 and FGFR1 overexpression, respectively. Concerning the *FGFR1* amplified primary tumors (n=5) only one harbored a *RAS* mutation. Both *FGF 3,4,19* amplified primary tumors were *RAS* wildtype (Table 2). In primary tumors which harbored more than one FGFR aberration a significantly higher frequency of *RAS* mutations could not be recognized in comparison to those with single changes.

## DISCUSSION

Colorectal cancer is one of the leading causes of cancer related deaths worldwide. According to the Surveillance, Epidemiology and End Results (SEER) DataBase the incidence of colorectal cancer is 135,430 in the US for 2017 and more than 50,000 patients are estimated to die of this disease annually. 39% of colorectal tumors are diagnosed in a localized stage and have an excellent prognosis with a 5-year-relative-survival of 89.9%. Even if the tumor has metastasized to the regional lymph nodes, the 5-year-survival is still 71.3% if an adequate therapy is applied. However, the occurrence of distant metastases defines a clinically fatal event in many cases. In a metastatic state, accounting for 21% of all CRC patients, the 5-year-relative-survival rate drops dramatically to 13.9% (SEER Database). Despite of multimodal up-to-date treatment regimens consisting of local intervention including surgery of metastases, chemotherapy in combination with monoclonal EGFR and VEGF antibodies, many patients still experience tumor recurrence and have a poor outcome. Novel innovative, personalized therapies may contribute to improved treatments. Oligometastatic patients with a limited number of liver metastases form a distinct clinical subgroup of CRC patients. Clinical outcome varies strongly within this group and efficient therapies are limited. Many of these patients have already received chemotherapy with or without antibody treatment prior to metastatic disease. Some of them can be cured by surgery or benefit from continuation of systemic treatment but others suffer from a progressive and often deadly disease. Currently, biomarkers have not yet been comprehensively established which allow prognostication and therapy selection in this clinically relevant subgroup.

All data that have been published on FGFR aberrations in CRC so far focused solely on primary tumors. Only few publications have described alterations of *FGFR1* [39, 40] or other *FGFR* genes [41], [42] up to now which are basically in line with our findings in primary colorectal cancers. However, in a very recent publication no gene amplifications of *FGFR 1-4* have been found [43]. A systematic overview over FGF/FGFR alterations in CRC has not yet been published.

In this study, we provide the first comprehensive data set on the prevalence of alterations in the *FGFR1-4* genes and the FGF ligands *FGF3,4* and *19* in oligometastatic CRC patients. We demonstrate that both gene amplification and overexpression occur in a subset of these patients. Moreover, we provide first evidence that FGFR3 overexpression (measured by RNA *in situ* hybridization in metastatic tissue) defines a specific subgroup with a significantly worse outcome (median overall survival 19.9 vs. 47.4 months, hazard ratio 3.14). In contrast, FGFR3 overexpression in primary tumors was not correlated with survival even if patients had synchronous distant metastases. Although only rarely observed, *FGFR1* amplification was significantly associated with a shortened overall survival in oligometastatic patients (median OS 12.6 vs. 47.4 months, HR=8.83).

In contrast to other tumor entities we could not demonstrate any FGFR gene fusions in our cohort. This may indicate that these changes play a minor role in colorectal cancer. However, we detected a number of gene copy number changes along with RNA overexpression. In our cohort, we could demonstrate *FGFR1* gene amplification, FGFR1 and FGFR3 overexpression in 4.8%, 8.5% and 14.9% of the liver metastases, respectively. FGFR2 overexpression and *FGF3,4,19* gene amplification were seen in primary tumors but not in our series of metastases indicating that these changes play probably a minor role in metastatic disease. Occasionally, tumors showed even multiple aberrations in the FGFR axis. We observed one case with multiple metachronous metastases where only the most recent lesions were FGFR3 positive. This might point towards the fact that FGFR3 overexpression may also evolve during tumor progression under treatment.

FGFRs and their ligands constitute a group of potential therapeutic targets in human cancers. A number of selective and non-selective tyrosine kinase inhibitors are currently studied in clinical trials in various tumor entities. Since FGFR signaling can be activated by different mechanisms, i.e. activating gene mutations, translocations, amplifications, overexpression or a combination of alterations, selection of the most appropriate predictive biomarker is crucial. To the best of our knowledge, activating *FGFR* mutations have never been described to play a significant role in colorectal cancer so far. Therefore, we focused on gene amplification and mRNA overexpression of FGF receptors and selected ligands which are most likely to occur in carcinomas and to represent potential therapeutic targets also in colorectal cancer. In our work, we studied extensively the prevalence of FGFR and FGF3,4,19 alterations in primary CRC and metastatic lesions and describe methods of biomarker evaluation. The most frequent change was an overexpression of FGFR3 which has not yet been described in this entity. This change was directly measured in tumor cells by applying an *in situ* approach. The used technology is readily applicable to clinical samples under routine conditions and is probably superior to immunohistochemistry. We decided against the usage of immunohistochemistry since stainings with currently available antibodies provided predominantly disappointing results and do not reach the level of quality and standardization required for routine applications. In a well conducted comprehensive study on FGFR3 alterations in bladder cancer Guancial et al. failed to demonstrate any clinically meaningful correlation of FGFR IHC. The authors themselves conclude that IHC staining does not appear to have prognostic or predictive value [44]. Therefore we preferred to apply RNA-ISH, a novel technique, which has also been used for patient selection in a recent phase I trial [34]. The preliminary response data from that early trial together with our findings on prevalence and prognostic impact make FGFR3 overexpression a potential therapeutic target also in metastatic colorectal cancer. Especially those oligometastatic CRC patients with worse prognosis might benefit from an anti-FGFR3 treatment. Therefore, we provide a possible rationale for future clinical research in that field and suggest including this subgroup in upcoming clinical trials with such drugs.

Limitations of our study were its retrospective nature and the small number of analyzed cases. Subsequent studies and larger cohorts will be required to confirm our results. These upcoming studies should also include further locations of metastases such as e.g. lung, since also the hepatic tumor environment comprising hepatocytes, stellate cells and Kupffer cells might have an influence on tumor behavior. However, in this study we intended to focus on liver metastases of oligometastatic CRC in order to present a rather homogenous cohort.

Furthermore, FGFR changes, especially FGFR3 overexpression are likely to occur also in more advanced colorectal cancers which we did not include in our investigation, i.e. tumors with metastases at multiple sites. Thus, we suggest analyzing these cases in future studies, too. In this study tumors and metastases were analyzed by using tissue microarrays. Therefore, we suppose that the number of cases with FGFR alterations might be even higher due to intratumoral heterogeneity which we did not fully capture.

Despite these limitations we provide first evidence that FGFR aberrations might represent potential therapeutically tractable events in a subset of colorectal cancer patients with FGFR3 mRNA overexpression being the most frequent alteration. The latter change, if measured in metastatic tissue, defines a subgroup with poor outcome in metastatic CRC. Thus, our data might serve as a basis for future clinical trials with FGFR-targeted therapies.

## MATERIALS AND METHODS

### Patients’ samples

This study has been carried out with tumor tissue of 195 patients. We investigated two cohorts: i) 140 primary colorectal tumors, thereof 70 colonic and 70 rectum carcinomas; ii) 63 liver metastases of CRC (55 patients, among them three with multiple lesions). Primary tumors and metastases originated from different patients. All samples derived from surgical specimens of either primary cancers or metastatic lesions and have been diagnosed by experienced pathologists. Metastases have been collected between 2011 and 2014 from liver surgery of oligometastatic patients. The patient cohort was characterized in terms of demographics, clinical baseline data, and treatment regimens. Follow-up examinations were performed according to individual physicians’ discretion and data were obtained either from the local clinical cancer registry or the treating physician. Overall survival (OS) after primary surgical treatment (OS primary tumor) was defined as the interval between the surgical resection of the primary tumor and cancer-related death. Baseline patients’ characteristics are summarized in Supplementary Table 1.

All tumor samples were examined by using tissue microarrays (TMAs) with a core needle diameter of 1 mm. This project was approved by the local ethics committee (application number 21/3/11).

### Fluorescence *in situ* hybridization (FISH)

All tumor samples were analyzed with fluorescence *in situ* hybridization (FISH) using a set of appropriate FISH probes for the detection of gene amplifications and chromosomal translocations (ZytoLight SPEC *FGFR1*/CEN 8 Dual Color Probe, ZytoLight SPEC *FGFR2*/CEN10 Dual Color Probe, ZytoLight SPEC *FGFR3*/CEN4 Dual Color Probe, ZytoLight SPEC *FGF3,4,19*/CEN 11 Dual Color Probe, ZytoLight SPEC *FGFR1* Dual Color Break Apart Probe (ZytoVision, Bremerhaven, Germany), Poseidon *FGFR4* (5q35)/5q11.2 Probe (Kreatech, Amsterdam, Netherlands), Agilent Sure FISH *FGFR3* (BA) probe (Agilent Technologies, CA, USA)). Hybridization and evaluation were performed as previously published [45]. Sixty tumor cell nuclei were analyzed in each tumor by counting green and orange signals of gene amplification probes. Break apart probes for the detection of rearrangements were evaluated in 50 contiguous tumor cell nuclei where tumors with ≥15% split orange and green signals were considered positive as previously described [46, 47]. In 10 cases with insufficient tissue or signal quality we accepted a minimum of 20 evaluable tumor cells.

In terms of gene amplification assays tumors were evaluated by applying a scoring system which has been established previously [45] and categorized into following groups:

i) high-level amplification was defined as
  a) target gene/centromere ratio ≥2.0 or
  b) average target gene copy number per cell of ≥6.0 or
  c) the percentage of tumor cells containing ≥15 target signals or large clusters is ≥10%;
ii) low-level amplification was defined as the percentage of tumor cells containing ≥5 target gene signals is ≥50%
iii) all other tumors were classified as negative.

### RNA *in situ* hybridization (RNA ISH)

RNA *in situ* hybridization for semi-quantitative determination of mRNA expression levels of FGFR1-3 was performed by using the ACD RNAscope Assay red detection kit (Advanced cell diagnostics, CA, USA) following the protocols suggested by the manufacturer. Briefly, 4μm thin formalin-fixed and paraffin-embedded TMA sections (FFPE) mounted on superfrost plus slides (Menzel Gläser, Thermo Scientific, Germany) underwent pretreatment with heat and protease. Afterwards, FGFR1-3 specific RNA probes were hybridized to the target RNA. Scoring of FGFR mRNA expression has been made according to the manufacturer’s recommendations: score 0, no staining or less than 1 dot to every 10 cells (40x magnification); score 1, 1-3 dots/cell (visible at 20-40x magnification); score 2, 4-10 dots/cell, very few dot clusters (visible at 20-40x magnification); score 3, >10 dots/cell, less than 10% positive cells have dot clusters (visible at 20x magnification); score 4, >10 dots/cell, more than 10% of signals are organized in clusters (visible at 20x magnification) [48]. On the basis of these scores the H-score is calculated according to the following equation: Score 0*0 + score 1*1 + score 2*2 + score 3*3 +score 4*4. Thus, the result ranged from 0 to 400 maximum, whereas mRNA overexpression was defined by an H-score of ≥200. Besides that, we also applied the predominant score as a criterion for mRNA overexpression with score ≥3 defining mRNA overexpression. The H-score and the predominant score were correlated afterwards. For subsequent statistical analysis, cases were considered as “RNA overexpression positive” based on the H-score with the cut-off at ≥200.

### Molecular subtyping of cases

All primary tumors were analyzed for *KRAS* exon 2 mutations. For metastases, molecular analyses of *KRAS*, *NRAS* and *BRAF* were carried out by using the Qiagen therascreen kits (therascreen *BRAF* Pyro Kit, therascreen *RAS* Extension Pyro Kit, therascreen *KRAS* Pyro Kit, therascreen *NRAS* Pyro Kit) as previously described [49]. *PIK3CA* was analyzed by applying high resolution melting analysis (HRM), mutations were confirmed by Sanger sequencing as described beforehand [50]. Mismatch repair deficiency/microsatellite instability (MSI) was evaluated by means of immunohistochemistry following the locally established protocol with the ready to use antibodies MLH1 (Clon ES05), MSH2 (Clon FE11), MSH6 (Clon Epi 49) and PMS2 (Clon EP51) (Dako, Agilent technologies, Glostrup, Denmark).

### Statistical analysis

FGFR aberration numbers in primary tumors were visualized with the R package 'VennDiagram' (version 1.6.17). Survival analysis on time-to-event data was conducted with the R package 'survival' (version 2.40) [51]. Kaplan-Meier curves of overall survival were compared using the log rank test. Data analyses were performed with the statistical computing software R, version 3.2.2 and with the SPSS 22.0 software (IBM, Armonk, NY, USA). Fisher’s Exact and chi-square test were applied to compare FGFR positive and FGFR negative cases with regard to clinicopathologic parameters and molecular subtypes. *T*-tests were applied to compare the age of patients with FGFR positive and negative lesions. All tests were two-sided; p<0.05 was considered positive.

## SUPPLEMENTARY MATERIALS TABLE



## Abbreviations

(av)GCN: (average) Gene Copy Number
CEN: Centromere
CI: Confidence Interval
CSF-1: Colony Stimulating Factor-1
EGFR: Epidermal Growth Factor Receptor
FFPE: Formalin Fixed and Paraffin Embedded
FGF(R): Fibroblast Growth Factor (Receptor)
FISH: Fluorescence *in situ* hybridization
HPSGs: Heparan Sulfate Proteoglycans
HR: Hazard Ratio
HRM: High Resolution Melting (Analysis)
(K)RAS: (Kirsten) Rat Sarcoma
LM: Liver Metastasis
MAPK: Mitogen-activated Protein Kinase
(m)CRC: (metastatic) Colorectal Cancer
(m)OS: median Overall Survival
(m)RNA: (messenger) Ribonucleic Acid
MSI: Microsatellite Instability
NSCLC: Non-Small Cell Lung Cancer
PDGFR: Platelet Derived Growth Factor Receptor
PI3K: Phosphoinositide-3-Kinase
PIK3CA: Phosphatidylinositol-4,5-Bisphosphate 3-Kinase Catalytic Subunit Alpha
RNA ISH: RNA *in situ* hybridization
SEER (Database): Surveillance, Epidemiology and End Results
STAT: Signal Transducers and Activators of Transcription
TMAs: Tissue Microarrays
VEGFR: Vascular Endothelial Growth Factor Receptor
WT: Wild Type
