# Supplementary material for: FGFR3 mRNA overexpression defines a subset of oligometastatic colorectal cancers with worse prognosis
Source: Oncotarget. 2018 Aug 14;9(63):32204–18. doi: 10.18632/oncotarget.25941 (PMC6114946; doi:10.18632/oncotarget.25941)
Supplement: Supplementary file 1 [file oncotarget-09-32204-s001.pdf]

FGFR3 mRNA overexpression defines a subset of oligometastatic colorectal cancers with worse prognosis

SUPPLEMENTARY MATERIALS

Supplementary Table 1: Patients' characteristics

		Primary tumors (n=140)	Metastases (n=55)
Gender (%)	Male	81 (57.9)	36 (65.5)
	Female	56 (40)	19 (34.5)
	Data not available	3 (2.1)	--
Age (Years)	Median	74	64
	Range	43-98	32-84
	Date not available	3	--
Localization / Origin of primary tumor (%)	Colon	70 (100)	30* (100)
	Left-sided	33 (47.1)	10 (33.3)
	Right-sided	31 (44.3)	10 (33.3)
	Transverse	5 (7.1)	--
	Date not available	1 (1.4)	10 (33.3)
	Rectum	70 (100)	24* (100)
T stage (%)	1	6 (4.3)	
	2	29 (20.7)	
	3	87 (62.1)	
	4	15 (10.7)	
	Data not available	3 (2.1)	
N stage (%)	0	75 (53.6)	
	≥1	62 (44.3)	
	Data not available	3 (2.1)	
M stage (%)	0	104 (74.3)	
	1	33 (23.6)	55 (100)
	Data not available	3 (2.1)	
Treatment ≤6 months before Resection of metastases (%)	Chemotherapy	--	6 (10.9)
	Chemotherapy + anti-EGFR	--	12 (21.8)
	Chemotherapy + anti-VEGF	--	9 (16.4)
	Chemotherapy + anti-EGFR + anti-VEGF	--	1 (1.8)
	Data not available	--	27 (49.1)

T, tumor size; N, regional lymph nodes; M, distant metastases; anti-EGFR, epidermal growth factor receptor antibodies; anti-VEGF, vascular endothelial growth factor antibodies; *, one patient had a simultaneous colon (cecum) and a rectal carcinoma, hence it remains unclear which carcinoma caused the metastases.
